# Cancer and thrombosis: recent advances

**DOI:** 10.3747/co.v15i6.296

**Published:** 2008-12

**Authors:** S. Solymoss

**Keywords:** Venous thromboembolism, risk factors, epidemiology, low molecular weight heparin

## INTRODUCTION

The burden of venous thromboembolism (vte) is a challenging problem in the medical management of cancer patients. The well-known association between 1 can now be rigorously studied with cancer and vte modern molecular techniques 2. Large population-based epidemiologic studies have shed light on the relative and cancer types 3,4, and clinical frequency of vte trials of cancer therapy have defined additional risk factors that compound the thrombotic risk 4–6.

## VTE AND CANCER PATIENTS

The treatment of cancer patients for vte has been associated with a high risk of recurrent thrombosis and of bleeding complications 7. However, recent data point to improved patient outcome with the use of long-term low molecular weight heparin therapy 8,9. Cancer patients who also have vte are at increased risk of mortality 10, but there are intriguing suggestions concerning the benefit of low molecular weight heparin for improved cancer survival 11. A recently published guideline facilitates the practice of evidence-based vte prevention and treatment in cancer patients, and sets the stage for future directions in this important domain 12.

### Attendant Problems

The inherent properties of blood vessels, endothelial cells, adhesion receptors, and soluble plasma coagulation proteins render these structures and molecules important in the propagation of coagulation and the regulation of cancer-cell growth. Perturbation of these components by cancer treatment interventions such as surgery, chemotherapy, and supportive care can further exacerbate prothrombotic properties.

Advanced cancer is more often associated with vte, as are certain cancer types, including pancreatic, stomach, brain, lung, and hematologic malignancies. In cancer patients, vte is not only a frequent cause of death, it also identifies a patient population with poor prognosis, and it is both clinically challenging and financially costly to treat. As cancer treatment modalities evolve, differences in the added thrombogenic risk for patients also emerge—for example, the addition of anti-angiogenic therapy is associated with one of the highest risks of thrombotic complications.

### Optimizing Treatment

Given the strong association of cancer and vte, how should thromboprophylaxis for cancer patients be optimized?

Clearly, as recently reviewed in *Current Oncology,* patients undergoing cancer surgery and those hospitalized for investigations and treatment of cancer are good candidates for appropriate thromboprophylaxis 13. Because all currently used anticoagulants are inherently associated with a risk of bleeding and because they generate added cost, prophylaxis of cancer patients at lower thrombotic risk cannot be justified at this time. Probing the role of low molecular weight heparins in modifying cancer progression should be the subject of additional well-designed clinical trials. All cancer-associated vte should be considered for long-term low molecular weight heparin therapy, given the more favourable clinical outcomes seen as compared with the outcomes seen with traditional oral anticoagulant therapy.

The recent guidelines from the American Society of Clinical Oncology underline the importance of optimizing anticoagulation management of cancer patients. The practice of appropriate thromboprophylaxis, up-to-date anticoagulation of cancer-associated vte, and participation in good clinical trials evaluating cancer and coagulation should routinely be part of our comprehensive care of cancer patients.
